# Hydro-Methanolic Extract of *Cornus Mas* L. and Blood Glucose, Lipid Profile and Hematological Parameters of Male Rats

**DOI:** 10.5812/ircmj.17784

**Published:** 2014-05-05

**Authors:** Bita Abdollahi, Mehran Mesgari Abbasi, Parvin Zakeri Milani, Ashraf Sadat Nourdadgar, Seyyed Mehdi Banan Khojasteh, Vahid Nejati

**Affiliations:** 1 Drug Applied Research Center, Tabriz University of Medical Sciences, Tabriz, IR Iran; 2 Student Research Committee, Tabriz University of Medical Sciences, Tabriz, IR Iran; 3 Liver and Gastrointestinal Research Center, Tabriz University of Medical Sciences, Tabriz, IR Iran; 4 Medical Education Research Center, Tabriz University of Medical Sciences, Tabriz, IR Iran; 5 Department of Natural Sciences, Faculty of Natural Sciences, University of Tabriz, Tabriz, IR Iran; 6 Faculty of Science, Urmia University, Urmia, IR Iran

**Keywords:** *Cornus*, Biological Markers, Hematologic Tests, Blood Glucose

## Abstract

**Background:**

*Cornus mas* L, an olive-shaped red-colored single-seeded fruit, is used in traditional medicine in different parts of Europe and Asia.

**Objectives:**

In the present study, 40 male Wistar rats were randomly divided into five groups, and the effects of 21 days of intraperitoneally (IP) administration of 50, 200 and 400 mg/kg body weight of *C. mas* hydro-methanolic extract on the rats hematological and biochemical parameters were investigated. The experimental study was carried out in Tabriz, Iran.

**Materials and Methods:**

The hematology and biochemical tests were performed by the Technicon H1 Hematology Analyzer and enzymatic methods, respectively.

**Results:**

The results indicated that all doses of the extract caused significant (P < 0.05) decreases in the hemoglobin distribution width (HDW) (2.3 ± 0.2 vs. 2.5 ± 0.2, P = 0.049) and platelet distribution width (PDW) (56.5 ± 1.8 vs. 63.9 ± 3.6, P = 0.001) of the treated groups vs. control group, whereas only high doses caused significant elevation in the mean corpuscular hemoglobin concentration (MCHC) (30.3 ± 0.8 vs. 28.6 ± 0.6, P = 0.047), mean platelet volume (MPV) (5.0 ± 0.6 vs. 4.1 ± 0.3, P = 0.002), total platelet mass (PCT) (0.33 ± 0.07 vs. 0.26 ± 0.01, P = 0.050), and significant decrease in the red cell distribution width (RDW) (13.8 ± 0.4 vs. 14.7 ± 1.3, P = 0.048) of the treated groups vs. control group.

**Conclusions:**

Decreasing effect of the extract on platelet activity might classify it as an alternative for antiplatelet therapy in cardiovascular diseases (CVD). The results of this study suggested that further investigations with higher doses of *C. mas* fruit extract are necessary to obtain significant protective and nonprotective changes in hematological and biochemical parameters.

## 1. Background

Cornelian cherry (*Cornus mas* L.) is the most important fruit from the 40 species of the Cornaceae family (1). *C. mas L*. is a species of dogwood, native to southern Europe and Asia (2-4). In Iran, cornelian cherry trees are spread in the west parts of the country (East Azerbaijan and Qazvin provinces) (1, 5). It is a medium-to-large deciduous shrub or small tree that grows up to 5 – 12 m. Cornelian cherries are typically olive-shaped single-seeded fruits, and 10-23 mm long, originating from an inferior ovary. They are typically red, but can also be found in pink and yellow in general, and are sweet-sour (1, 4, 6, 7). It is worth noting that different products are produced from the cornelian cherry. The fruits not only are consumed fresh but also used to produce jam, stewed fruit, compote, syrup, and several types of soft drinks. In Iran, cornelian cherry fruits are consumed freshly, dried whole, and pickled like olives (1).

There are some investigations regarding the physical and chemical properties of cornelian cherry fruits (3, 8, 9). Fresh cornelian cherry fruits, containing twice as much ascorbic acid (vitamin C) as oranges, show their potential as a food additive (1, 4, 6, 7). These fruits are also rich in sugar, organic acids, tannins, anthocyanins, phenols, and other antioxidants (8, 10, 11). Compared to other juices obtained from plum, pear and apple, cornelian cherry juice contains high levels of Calcium, reaching 10 folds higher (323 mg/L) than other juices (14-77 mg/L). Furthermore, cornelian cherries have high contents of K and Mg, but are low in Na and other essential minerals (Cu, Mn, Fe, and Zn); their levels of toxic elements are also negligible (9-14). In Iranian, Caucasus and Central Asia traditional medicine, cornelian cherry was being used for more than 1000 years (11, 15, 16). Galenicals are made from leaves and flowers, and fruits have been used to treat sore throat, digestion problems, measles, chickenpox, anemia, rickets, and liver (hepatitis A) and kidney (pyelonephritis) diseases in traditional and conventional medicine (15, 17, 18). The juice and the evaporated juice of the fruit are used against diabetes as well (19, 20). Besides, the galenicals from leaves, dried fruits powder, ground, and dried drupes are widely used in diarrhea and hemorrhoids treatments (15, 17). The fruit flesh and the seed oil are successfully used for curing difficult-to-heal wounds, stomach ulcers, and colitis (15, 17, 18, 21). Moreover, several *cornus* spp. fruits are used to improve liver and kidney functions. It was also reported to have antibacterial, antihistamine, antiallergic, antimicrobial, and antimalarial activities (3). In this respect, the astringent property of this fruit is a good treatment for bowel complaints and fevers, and also used in cholera treatment (3). Scholars in the former Soviet Union noted that the fruit flesh and the seed oil were also useful for recovery and regeneration of damaged inner and outer epidermic tissues (skin and mycoderms) (15, 17, 18).

Furthermore, *C. mas*, a medicinal plant, was mentioned for treatment of circulation disorders and blood dilution in traditional medicine in Iran. However, this plant has not been subjected to systematic investigation to assess its hematological and biochemical effects. Reviewers believe that the researched to date have had poor qualities, and larger and more rigorous trials are needed to investigate the possible adverse effects associated with excessive polyphenol intake by consuming polyphenol-rich fruits; currently, lack of knowledge about the polyphenol safety, suggests that its levels should not exceed a normal diet amount (22). Moreover, hematological parameters assessment can be used to determine the extent of deleterious effects of the extracts on the animal blood. It can also be used to explain the blood-related functions of the plant extract or its products (23).

## 2. Objectives

Aim of the present study was to investigate some hematological and biochemical effects of *C. mas *fruit extract on healthy male rats.

## 3. Materials and Methods

### 3.1. Plant Ingredients and Extraction

The *C. mas* fruits were obtained from the suburbs of Kaleibar (East Azarbaijan, Iran). The fruits were washed and their seeds were removed. The fruit plant parts were air-dried, protected from direct sunlight, and then turned into a coarse powder. Afterwards, 500 g of the powder was extracted by methanol and water mixture (7:3) at 25 ± 2°C. The mixture was then filtered and the solvent was completely removed by a rotary vacuum evaporator at 50°C. Finally, the *C. mas* fruits extract was frozen, dried and stored until use.

### 3.2. Animals

In this experimental study, 40 male Wistar rats, weighing 220 ± 20 g, were used. The animals were housed in polycarbonate standard cages in a temperature-controlled room (22 ± 2°C) under 12/12 hours of light/dark cycles for one week, before and during the experiments. Next, the animals were provided by a standard rat pellet diet and clean drinking water ad libitum. All the ethical and humanity considerations as well as euthanasia of the animals were considered and performed during the experiments.

### 3.3. Procedures

The sample size was determined according to the previous similar animal studies (24-27). Using the Randlist software, the animals were randomly divided into five groups (eight animals in each group) as follows:

1. Group I served as the normal control and received a normal diet without any injection.
2. Group II served as the placebo control and intraperitoneally (IP) received a normal saline, daily for three weeks.
3. Group III-V served as the treatment groups. They IP received the *C. mas* fruit extract (CMFE) at the doses of 50, 200 and 400 mg/kg of body weight (BW), daily for three weeks.

At the end of the interventional period, the blood samples were taken from each animal by cardiac puncture. Samples were immediately transferred into tubes together with ethylenediaminetetraacetic acid (EDTA) as the hematology anticoagulant to analyze and clot the tubes for biochemical analyses. All the animals experiments were approved by the Research Ethics Committee of Tabriz University of Medical Sciences (ethical approval code: 5-4-1171, date: 4 May 2013) and performed according to the Helsinki’s humanity research declaration.

### 3.4. Hematological Analyses

The hematology parameters were explored by the Technicon-H1 (Bayer) hematology analyzer. Technicon H-1 apparatuses are discrete analyzers that perform complete blood and platelet counts, as well as leukocyte differential count. The instrument has a tungsten halogen light source, a cytometer for leukocyte peroxidase analysis, and a helium-neon red laser for RBC/platelet and basophil determinations. In addition, within the cell flow, the cells were classified one by one based on size and cytochemical peroxidase reaction. The instrument was calibrated and validated using several calibrators before testing the samples. The results of the analyzer were checked by microscopic analyses of the blood smears, stained with May–Grünwald and Giemsa–Romanowski.

### 3.5. Biochemical Analyses

The clotted blood samples were centrifuged at 2000 g, and the blood serums were separated and placed in -70ºC freezer until performing the tests. Afterward, the blood glucose, triglyceride, cholesterol, low density lipoprotein (LDL), and high density lipoprotein (HDL) were determined by the enzymatic methods (Parsazmun, Iran) with the automated Abbott, Alcyon 300 biochemistry analyzer (USA). The instrument was calibrated and validated using several calibrators before testing the samples.

### 3.6. Data Analyses

Before presenting the results, Kolmogrof-smirnof test was performed with Q-Q chart for surveying the normality. In addition, results of Levene test showed equality of variances of the groups. All the results were expressed as mean ± SD. One-way analysis of variance (ANOVA) was used to compare different parameters between the groups, followed by multiple comparisons with the Tukey post-hoc test. P value < 0.05 was considered significant.

## 4. Results

There were no significant differences in the red blood cell (RBC) count, hemoglobin (Hb), packed cell volume (PCV), mean corpuscular volume (MCV), and mean corpuscular hemoglobin (MCH) between the treated and control groups (Table 1). However, a significant increase (P = 0.047) in the mean corpuscular hemoglobin concentration (MCHC) was found in the *C. mas* high dose (400 mg/kg BW) treated group in comparison with the control group (Table 1). There were also significant decreases in red cell distribution width (RDW) in the groups with 200 and 400 mg/kg BW. Besides, there were significant decreases (P = 0.049) in hemoglobin distribution width (HDW) in the groups treated with 50, 200, and 400 mg/kg BW *C. mas*, compared with the control groups (Table 1).

**Table 1.** Red Blood Cell Parameters of the *Cornus mas* Fruit Extract-Treated Rats ^a,b^

Parameter/Group	Control (Normal)	Control (Placebo)	*C. mas* (50 mg/kg BW)	*C. mas* (200 mg/kg BW)	*C. mas* (400 mg/kg BW)	P Value
**RBC, 10^12^/L**	7.8 ± 0.6	7.8 ± 0.6	7.6 ± 0.3	7.7 ± 0.4	7.9 ± 0.5	0.582
**Hb, g/dL**	13.0 ± 0.5	13.1 ± 0.8	13.1 ± 0.9	12.9 ± 0.4	13.5 ± 0.9	0.407
**PCV, %**	45.6 ± 2.1	45.4 ± 2.1	44.1 ± 2.1	43.4 ± 2.0	46.3 ± 5.6	0.364
**MCV, fL**	58.1 ± 2.3	58.0 ± 2.4	58.6 ± 2.6	56.4 ± 1.5	56.7 ± 1.7	0.163
**MCH, pg**	16.6 ± 0.9	16.6 ± 0.8	17.3 ± 0.9	16.8 ± 0.7	17.2 ± 0.5	0.339
**MCHC, g/dL**	28.6 ± 0.6	28.8 ± 0.9	29.6 ± 1.3	29.7 ± 1.3	30.3 ± 0.8	0.047
**RDW, %**	14.7 ± 1.3	14.7 ± 0.9	13.9 ± 0.7	13.9 ± 0.5	13.8 ± 0.4	0.048
**HDW, g/dL**	2.5 ± 0.2	2.5 ± 0.2	2.3 ± 0.1	2.3 ± 0.2	2.3 ± 0.1	0.049

^a^ Data are expressed as mean ± SD, the parameters with significant differences (P < 0.05).

^b^ Abbreviations: Hb, hemoglobin; MCH, mean corpuscular hemoglobin; MCHC, mean corpuscular hemoglobin concentration; MCV; mean corpuscular volume; RBC, red blood cell count; PCV, packed cell volume; RDW, red cell distribution width.

Although results of the present study showed no significant differences between many of the RBC and hemoglobin-dependent parameters, Table 1 shows that treatment with the high-dose (400 mg/kg BW) CMFE caused a significant increase (P < 0.05) in MCHC. That is due to the nonsignificant decrease of MCV and increase of MCH in this group in comparison with the control group. MCHC might be expected to increase in the treated rats with higher doses of CMFE; it is suggested to be studied in future. The results also indicated that CMFE caused a decrease in RDW that was significant (P = 0.048) in 200 and 400 mg/kg BW doses. RDW is the the frequency distribution curve width of the RBC volume (one SD) divided by the mean RBC volume. Therefore, decrease of RDW meant that the RBCs did not vary much in size (28); it was probably resulted from the decrease (nonsignificant) of MCV. In addition, there was a significant decrease (P = 0.049) in HDW in the treated groups in comparison with the control groups. This meant that the hemoglobin contents of the RBCs did not vary much, probably resulted from nonsignificant increase of MCH. Significant increase of MCHC and decrease of RDW were in agreement with the effects of antioxidant-rich natural compounds on hematopoiesis (29-32). The results showed that treatment with 400 mg/kg BW of CMFE caused a significant increase (P = 0.002) in the mean platelet volume (MPV) and total platelet mass (PCT). There was also significant increase in MPV in the group treated with 200 mg/kg BW of CMFE (Table 2). A significant decrease (P = 0.001) in the platelet distribution width (PDW) was also found in the groups treated with 50, 200, and 400 mg/kg BW of CMFE (Table 2). The high dose (400 mg/kg BW) caused nonsignificant increase in the platelet count as well. The abovementioned changes in the platelet-related parameters suggested platelet activity inhibition without bone marrow suppression in the treated groups in comparison with the control groups. MPV and PDW increased during the platelet activation. PDW is a more specific marker of the platelet activation, since it does not increase during the simple platelet swelling (33). In this regard, increased MPV indicated a large number of larger and younger platelets in the blood, probably due to the bone marrow rapid production and release of the platelets into the blood circulation. Besides, decreased PDW indicated uniformity in the size of platelets and inactivation of the platelets, which was in agreement with traditional medicine specialists’ believes about the cardiovascular protective effects of *C. mas*. It is evident that antioxidants reduce the platelet activity through stimulation of prostacyclin synthesis and scavenging the synthesis-inhibiting peroxides. The experimental and clinical evidences supporting this theory are inconclusive (34, 35). Furthermore, recent studies have shown that polyphenols such as catechin and quercetin, stop NADPH oxidase enzyme (present in platelets). Therefore, inhibiting the production of O2¯ and increasing the biological power of NO consequently regulate glycoprotein pine receptors on platelet membrane surface (GPIIb-IIIa), that in turn inhibits platelets’ activation and their adherence during inflammation and thrombosis processes (36). As a result, it is probable that the antioxidant compounds in *C. mas* L. fruits, including vitamins, anthocyanins (37), and phenolic compounds, prevent the platelet activity. Platelet hyperactivity is one of the well-identified risk factors for cardiovascular disorders (CVD) developments and complications (38, 39). Moreover, antiplatelet therapies are associated with resistance; they were reported to have side effects and contraindications which were prompted for investigations of natural products as alternatives for prevention and treatment of CVD (40-42). Treating with *C. mas* may be a good choice for this purpose, but more investigations and trials are necessary.

**Table 2.** Platelet Parameters of the *Cornus mas* Fruit Extract-Treated Rats ^a,b^

Parameter/Group	Control (Normal)	Control (Placebo)	*C. mas* (50 mg/kg BW)	*C. mas* (200 mg/kg BW)	*C. mas* (400 mg/kg BW)	P Value
**PLT, 10^9^/L **	545.3 ± 38.5	531.1 ± 88.2	514.7 ± 123.3	545.3 ± 124.9	607.5 ± 124.5	0.449
**MPV, fL **	4.1 ± 0.3	4.1 ± 0.3	4.2 ± 0.3	4.6 ± 0.5	5.0 ± 0.6	0.002
**PDW, %**	63.9 ± 3.6	63.6 ± 3.2	62.4 ± 1.0	60.3 ± 4.4	56.5 ± 1.8	0.001
**PCT, %**	0.26 ± 0.01	0.24 ± 0.02	0.24 ± 0.05	0.29 ± 0.07	0.33 ± 0.07	0.050

^a^ Abbreviations: BW, body weight; *C. mas*, *Cornus mas;* MPV; mean platelet volume, PCT; total platelet mass, PDW; platelet distribution width.

^b^ Data are expressed as mean ± SD, the parameters with significant differences (P < 0.05).

The results showed no significant changes (P = 0.279) in white blood cells (WBC), absolute neutrophils (NEUT), lymphocytes (LYMP), monocytes (MONO), eosinophils (EOS) and basophils (BASO) counts, differential leukocyte percent, large unstained cells (LUC), myeloperoxidase index (MPXI), and lobularity index (LI) between the treated and control groups. There were not significant differences between the results of leukocytes related parameters (Table 3), whereas Table 3 shows mild continuous increases in the percentages, absolute counts of the eosinophils, and LUC, as well as mild decreases in the LI between the groups from low to high doses. In addition, significant increases in the eosinophils and LUC, and significant decreases in the LI, treating with higher doses than the ones used in this study might be expected. Moreover, increase in the eosinophils may be due to reduction in the eosinophil apoptosis. Eosinophil apoptosis is mediated by stimulators of cellular oxidative metabolisms and inhibited by antioxidants (43). The results suggest that antioxidant properties of CMFE may inhibit the eosinophil apoptosis. LUCs are large cells without myeloperoxidase (MPO). Large atypical lymphocytes, plasma cells, and some blasts are characterized as LUCs. The MPO inhibition, interaction activity of the polyphenols, and other antioxidants were also reported by some researchers (44, 45). Further investigations with different and higher doses of CMFE are necessary for discussing its effect on the MPO activity and the granules.

**Table 3.** White Blood Cell Parameters of *Cornus mas* Fruit Extract-Treated Rats ^a,b^

Parameter/Group	Control (Normal)	Control (Placebo)	*C. mas* (50 mg/kg BW)	*C. mas* (200 mg/kg BW)	*C. mas* (400 mg/kg BW)	P Value
**WBC, ×10^9^/L**	9.86 ± 2.13	11.26 ± 2.22	11.15 ± 2.44	9.73 ± 3.34	13.11 ± 5.58	0.279
**NEUT, ×10^9^/L**	1.36 ± 0.65	1.36 ± 0.65	1.32 ± 0.64	1.79 ± 0.86	1.86 ± 0.92	0.762
**NEUT, %**	12.8 ± 6.3	12.5 ± 5.8	11.3 ± 5.5	18.5 ± 9.2	13.6 ± 6.3	0.400
**LYMP, ×10^9^/L**	6.29 ± 1.33	7.84 ± 1.55	7.75 ± 1.67	6.00 ± 2.77	8.67 ± 3.88	0.206
**LYMP, %**	64.0 ± 6.2	63.9 ± 8.5	69.6 ± 4.5	61.1 ± 11.5	65.2 ± 10.4	0.389
**MONO, ×10^9^/L**	1.36 ± 0.56	1.29 ± 0.52	1.16 ± 0.48	1.04 ± 0.31	1.34 ± 0.66	0.606
**MONO, %**	14.9 ± 7.2	12.5 ± 6.1	11.1 ± 5.3	11.3 ± 4.0	11.3 ± 5.4	0.587
**EOS, ×10^9^/L**	0.16 ± 0.08	0.10 ± 0.05	0.11 ± 0.05	0.13 ± 0.06	0.20 ± 0.10	0.785
**EOS, %**	1.45 ± 0.72	0.95 ± 0.47	0.98 ± 0.48	1.53 ± 0.76	1.65 ± 0.81	0.868
**BASO, ×10^9^/L**	0.11 ± 0.04	0.15 ± 0.05	0.11 ± 0.05	0.08 ± 0.06	0.18 ± 0.88	0.084
**BASO, %**	1.21 ± 0.60	1.20 ± 0.52	0.95 ± 0.19	0.83 ± 0.30	1.33 ± 0.54	0.189
**LUC, ×10^9^/L**	0.59 ± 0.29	0.71 ± 0.34	0.68 ± 0.34	0.66 ± 0.32	0.83 ± 0.40	0.651
**LUC, %**	5.91 ± 2.10	5.81 ± 2.33	6.10 ± 2.40	6.73 ± 3.32	14.65 ± 7.20	0.531
**LI**	1.24 ± 0.05	1.24 ± 0.05	1.23 ± 0.06	1.22 ± 0.04	1.18 ± 0.05	0.199
**MPXI**	-40.3 ± 3.8	-40.2 ± 3.8	-40.2 ± 4.6	-38.8 ± 3.2	-39.0 ± 3.2	0.254

^a^ Abbreviations: BW, body weight; BASO, basophils; *C. mas*, *Cornus mas*; EOS, eosinophils; LYMP, lymphocytes; LUC, large unstained cells; LI, lobularity index; MONO, the monocytes; MPXI, the myeloperoxidase index; NEUT, the absolute neutrophils; WBC, white Blood Cell.

^b^ Data are expressed as mean ± SD, the parameters with significant differences (P < 0.05).

The results indicated no significant differences (P = 0.917) in the glucose, cholesterol, LDL, HDL, and triglyceride amounts, between the treated and the nontreated groups (Table 4). However, a mild decrease and a mild increase (but not significant) in the total cholesterol and HDL were found, respectively; it was based on findings of some previous studies (24). There were some reports about hypolipidemic effects of natural products anthocyanins (25). Higher doses of CMFE were expected to cause significant decrease and increase in cholesterol and HDL, respectively; it is suggested for future studies.

**Table 4.** Blood Serum Biochemical Parameters of *Cornus mas* Fruit Extract-Treated Rats ^a^

Parameter/Group	Control (Normal)	Control (Placebo)	*C. mas* (50 mg/kg BW)	*C. mas* (200 mg/kg BW)	*C. mas* (400 mg/kg BW)	P Value
**Glucose, mg/dL**	151.3 ± 48.9	152.5 ± 37.5	151.3 ± 35.3	167.4 ± 82.5	150.3 ± 12.0	0.917
**Cholesterol, mg/dL**	73.3 ± 18.0	72.4 ± 9.8	64.7 ± 6.4	64.4 ± 23.5	65.4 ± 9.1	0.719
**HDL, mg/dL**	25.3 ± 6.8	25.5 ± 6.4	27.3 ± 3.9	27.6 ± 5.1	26.7 ± 7.0	0.902
**LDL, mg/dL**	30.0 ± 4.2	29.3 ± 4.3	30.0 ± 3.6	32.5 ± 3.5	26.7 ± 3.5	0.872
**Triglyceride, mg/dL**	75.8 ± 16.6	84.6 ± 16.2	63.8 ± 10.5	74.6 ± 19.7	78.9 ± 12.0	0.320

^a^ Data are expressed as mean ± SD, the parameters with significant differences (P < 0.05).

Results of some previous studies showed that *C. mas* had antidiabetic effects and can improve the pancreas damage caused by free radicals in diabetic rats (26). However, there was a controversy about its hypoglycemic effect on healthy animal models (27). In addition, there were some interventional factors, probably interfered our detected parameters; one of them was nutritional deficiencies of rats. We used commercial laboratory animal food in our study. The food analysis report did not declare many microelement and vitamin quantities.

## 5. Discussion

Results of the present study indicated that all doses of *C. mas* hydro-methanolic extract caused significant decreases in HDW and PDW, whereas only high doses (200 and 400) caused significant elevation in MCHC, MPV and PCT, and significant decrease in RDW. Furthermore, trend of the results suggested the probability of *C. mas* usage as prophylactic and alternative therapy for CVD. However, further investigations with higher doses of CMFE are necessary to obtain significant protective and nonprotective changes in hematological and biochemical parameters.
